# Single Cell Profiling of Circulating Tumor Cells: Transcriptional Heterogeneity and Diversity from Breast Cancer Cell Lines

**DOI:** 10.1371/journal.pone.0033788

**Published:** 2012-05-07

**Authors:** Ashley A. Powell, AmirAli H. Talasaz, Haiyu Zhang, Marc A. Coram, Anupama Reddy, Glenn Deng, Melinda L. Telli, Ranjana H. Advani, Robert W. Carlson, Joseph A. Mollick, Shruti Sheth, Allison W. Kurian, James M. Ford, Frank E. Stockdale, Stephen R. Quake, R. Fabian Pease, Michael N. Mindrinos, Gyan Bhanot, Shanaz H. Dairkee, Ronald W. Davis, Stefanie S. Jeffrey

**Affiliations:** 1 Department of Surgery, Stanford University School of Medicine, Stanford, California, United States of America; 2 Stanford Genome Technology Center, Stanford University, Palo Alto, California, United States of America; 3 Department of Electrical Engineering, Stanford University, Stanford, California, United States of America; 4 Department of Health Research and Policy (Biostatistics), Stanford University School of Medicine, Stanford, California, United States of America; 5 BioMaPS Institute for Quantitative Biology, Department of Physics, Department of Molecular Biology and Biochemistry, Rutgers University, Piscataway, New Jersey, United States of America; 6 Department of Medicine, Stanford University School of Medicine, Stanford, California, United States of America; 7 Howard Hughes Medical Institute, Department of Bioengineering, Stanford University, Stanford, California, United States of America; 8 Cancer Institute of New Jersey, New Brunswick, New Jersey, United States of America; 9 Simons Center for Systems Biology, Institute for Advanced Study, Princeton, New Jersey, United States of America; 10 California Pacific Medical Center Research Institute, San Francisco, California, United States of America; University of California, Merced, United States of America

## Abstract

**Background:**

To improve cancer therapy, it is critical to target metastasizing cells. Circulating tumor cells (CTCs) are rare cells found in the blood of patients with solid tumors and may play a key role in cancer dissemination. Uncovering CTC phenotypes offers a potential avenue to inform treatment. However, CTC transcriptional profiling is limited by leukocyte contamination; an approach to surmount this problem is single cell analysis. Here we demonstrate feasibility of performing high dimensional single CTC profiling, providing early insight into CTC heterogeneity and allowing comparisons to breast cancer cell lines widely used for drug discovery.

**Methodology/Principal Findings:**

We purified CTCs using the MagSweeper, an immunomagnetic enrichment device that isolates live tumor cells from unfractionated blood. CTCs that met stringent criteria for further analysis were obtained from 70% (14/20) of primary and 70% (21/30) of metastatic breast cancer patients; none were captured from patients with non-epithelial cancer (n = 20) or healthy subjects (n = 25). Microfluidic-based single cell transcriptional profiling of 87 cancer-associated and reference genes showed heterogeneity among individual CTCs, separating them into two major subgroups, based on 31 highly expressed genes. In contrast, single cells from seven breast cancer cell lines were tightly clustered together by sample ID and ER status. CTC profiles were distinct from those of cancer cell lines, questioning the suitability of such lines for drug discovery efforts for late stage cancer therapy.

**Conclusions/Significance:**

For the first time, we directly measured high dimensional gene expression in individual CTCs without the common practice of pooling such cells. Elevated transcript levels of genes associated with metastasis *NPTN, S100A4*, *S100A9*, and with epithelial mesenchymal transition: *VIM, TGFß1, ZEB2, FOXC1*, *CXCR4,* were striking compared to cell lines. Our findings demonstrate that profiling CTCs on a cell-by-cell basis is possible and may facilitate the application of ‘liquid biopsies’ to better model drug discovery.

## Introduction

To cure epithelial-based cancers–such as cancers of the breast, prostate, lung, colon, and pancreas–therapies need to be directed toward those cells that cause metastases. Lethal epithelial cancers generally originate in a primary tumor and then spread (metastasize) to other organs by shedding cells into the bloodstream and/or lymphatic channels. Disseminating metastatic cells may lodge, remain dormant for varying amounts of time, and ultimately grow as secondary tumors in other body sites. Secondary tumors may re-seed additional metastatic cells into the bloodstream [1], [2], causing subsequent tumor spread that result in multiple metastatic tumors within the same organ and colonization of tumor cells in additional organs, generally leading to patient demise.

While considerable progress has been made towards elucidating the basic biology of primary tumors to guide therapy, the molecular characterization of metastatic disease, which generally occurs months or years after primary tumor excision, remains limited. The treatment of patients with metastatic disease continues to be based largely on biomarkers from their primary tumor, despite frequent discordance between primary and metastatic cancer [3], [4]. Some patients with disseminated disease may undergo biopsy of a single metastatic focus even though multiple foci are concurrently present. However, as the majority of metastatic lesions are never biopsied due to anatomic inaccessibility or associated morbidity of the procedure, they are unavailable for biological characterization. On the other hand, CTCs offer a readily accessible means of studying the biology of metastatic cells throughout the course of disease [5], [6], and are often referred to as a “liquid biopsy” [7].

CTCs are rare epithelial cells present in cancer patient blood amidst approximately 5×10^9^ anuclear red blood cells and 5–10×10^6^ nucleated white blood cells (leukocytes) per ml. Due to the general absence of epithelial cells in normal blood, the standard definition of a CTC is an epithelial cell found in the blood of a patient with cancer, confirmed by 1) visualization of an intact nucleus using DAPI, 4′,6-diamidino-2-phenylindole, a DNA-binding fluorescent stain; 2) expression of cytokeratin; and 3) lack of expression of the white blood cell marker, CD45, the leukocyte-common antigen gene [6], [8].

According to the current standard of care, which includes surgical resection of primary tumors, CTCs identifiable in the blood of patients with metastatic recurrence must, by definition, derive from metastatic foci. The number of CTCs in blood samples has been shown to correlate with clinical outcome in patients with metastatic breast, prostate, colorectal, and lung cancer [9]–[13]. Additional biological characterization of CTCs is confounded by significant leukocyte contamination or limited methodological sensitivity, thereby requiring sample pooling [14], [15]. To address this, we developed an immunomagnetic separation technology, the MagSweeper, that gently extracts live CTCs with high purity from unfixed, unfractionated blood, and facilitates robust analyses at the single cell level [16], [17].

Intratumoral heterogeneity of primary breast cancers is well illustrated by the presence of distinct oncogene mutations even within a single microscopic field of tumor tissue [4]. Such heterogeneity likely extends across the qualitative and/or quantitative expression of a multitude of genes resulting in distinctive molecular phenotypes of clonal metastatic lesions at different organ sites [18]. To prove the feasibility of high dimensional single cell analysis of CTCs and explore the magnitude of CTC heterogeneity across genes commonly known to be associated with breast cancer phenotypes, we transcriptionally profiled single CTCs isolated by the MagSweeper. We identified 2 major CTC subgroups in patients with primary and metastatic breast cancer. CTC subgroups appeared to cluster independently of established biomarkers observed in the primary tumor, such as ER, PR, and HER2 status. Heterogeneity among CTCs was significant, and cell-to-cell variations occurred even within a single blood draw. Our finding of CTC variability is consistent with primary and metastatic tumor heterogeneity and suggests that single cell phenotyping of CTCs is a practical approach to exploit this variability for the effective implementation of molecular guided cancer therapy on a more comprehensive scale than possible with mutational analysis of a few known genes.

## Materials and Methods

### Ethics Statement

This study was reviewed and approved by Stanford’s Human Subjects Research Compliance Board and adhered to HIPAA regulations. All human subjects signed informed consent prior to blood sample collection.

### Cell Culture

MCF7, SKBR3, T47D and MDA-MB-231 breast cancer cell lines were purchased from American Type Culture Collection (ATCC) and tested to be free of mycoplasma contamination. Since these cell lines were originally derived from disseminated lesions of the human host (www.atcc.org), they are designated as ‘metastatic’. Cells were cultured in Dulbecco’s Modified Eagle Medium high glucose supplemented with 10% fetal bovine serum (FBS) and 100 units per ml of Penicillin-Streptomycin (Invitrogen) and grown at 37°C and 5% CO_2_ in a humidified atmosphere. In addition, well-characterized, novel cell lines - CCdl054, CCdl672, CCdl675, previously developed from clinical primary breast tumor samples [19]–[21], were included in this study. Primary tumor cell lines were propagated in MCDB170 growth medium supplemented with 2% FBS as described earlier [22].

### Patient Samples

Study participants with primary and metastatic breast cancer were recruited through the Stanford Breast Oncology Clinic at the discretion of their treating medical oncologists. Blood was collected by venipuncture or from implanted venous access ports or both into 10 mL BD Vacutainer plastic EDTA tubes (Becton Dickinson). The first 9 ml tube of blood from each blood draw was discarded to prevent contamination by skin epithelial cells from the needle puncture site. Then, approximately 9 ml of blood was collected from each human subject and kept at room temperature. All blood samples were processed within three hours of collection.

### Circulating Tumor Cell Isolation using MagSweeper

**Figure 1:**
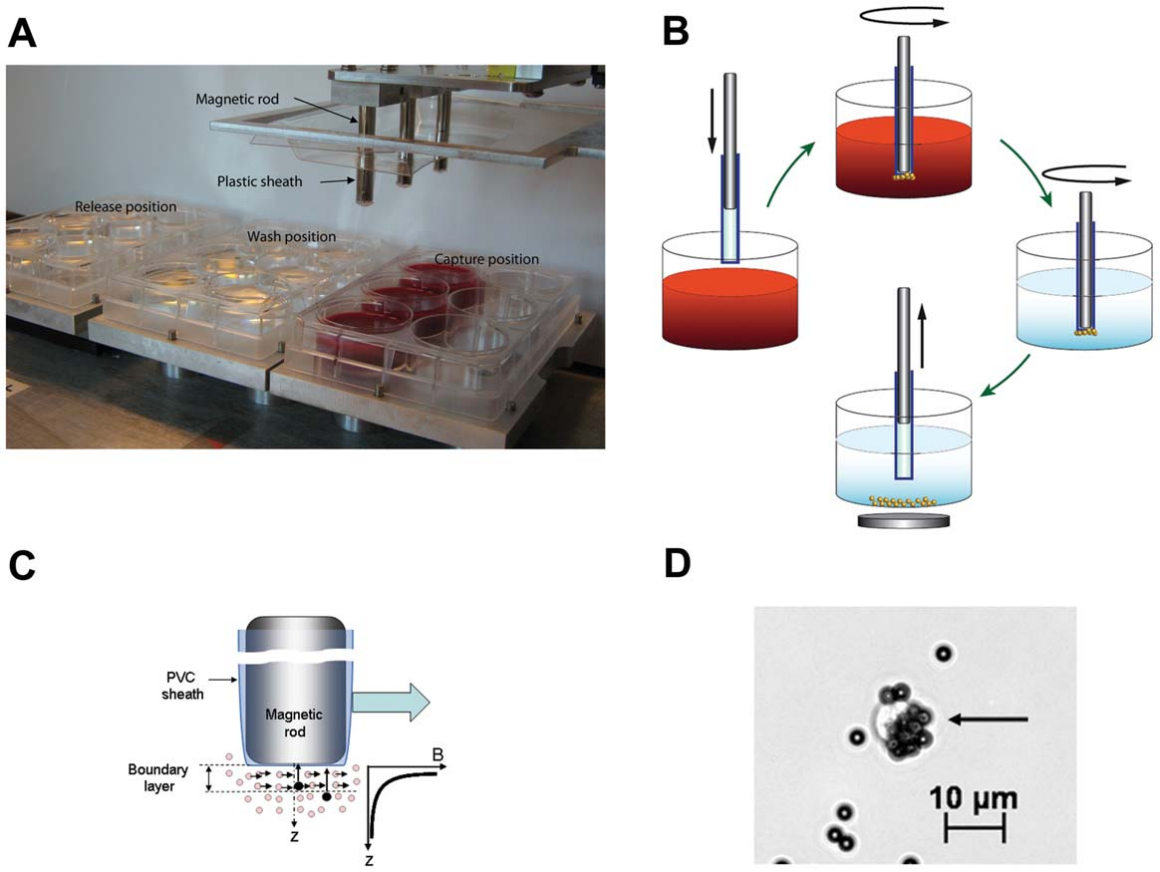
MagSweeper instrumentation, and cell isolation steps. A. MagSweeper device showing magnetic rods sheathed in plastic above the capture, wash and release stations. B. A diagrammatic view of MagSweeper cell isolation protocol. C. A controlled shear force produced by the movement of the magnetic rods in the wash station releases non-specifically bound blood cells. For cells with attached magnetic beads (black circles), the magnetic rod produces a magnetic force in z proportion to the nonuniformity (dB2/dz) of the magnetic field, thus imparting momentum in z proportional to (dB2/dz) and to a dwell time that depends both on the sweep speed and on the velocity distribution across the boundary layer that extends into the fluid from the surface of the sheath, optimizing capture of labeled cells and release of contaminating unlabeled cells. D. Photomicrograph (200X) of a CTC labeled with 4.5 µm immunomagnetic beads isolated from a patient with metastatic breast cancer. Magnetic beads are small dark spheres; the CTC appears as a translucent cell surrounded by clusters of beads.

To isolate CTCs, whole blood was labeled with 4.5 µm magnetic beads (Dynabeads Epithelial Enrich, Invitrogen) coated with the monoclonal BerEP4 antibody against human EpCAM (epithelial cell adhesion molecule, formally known as TACSTD1). Cells were labeled at room temperature with constant mixing for one hour. The samples were then diluted with PBS and processed for capture by a sweeping magnetic device - the MagSweeper (Figures 1A & 1B)**.** Two rounds of capture-wash-release were performed for all studies, whereby the movement of the magnet produced a controlled shear force that released many non-specifically bound leukocytes and other blood cells (Figure 1C). Captured cells were released into fresh buffer, then visually identified and photographed using an Axio Observer A1 inverted microscope (Carl Zeiss). Single cells were manually aspirated under visual guidance into a 1 µl volume using a Pipetman P2 (Gilson) (Figure 1D). The captured cells were then added to 0.2 µl of SUPERase-In RNAse inhibitor **(**Applied Biosystems/Ambion) and frozen on dry ice. Individual CTCs were stored at -80°C until analyzed.

### Preamplification

Single tumor cells contain picogram quantities of RNA, insufficient for reproducible whole genome microarray analysis. Target genes were preamplified using TaqMan gene expression assays (20x) (Applied Biosystems) and CellsDirect qRT-PCR kit (Invitrogen). The TaqMan gene expression assays (20x) were combined and diluted with TE (Tris and EDTA) buffer to yield 0.2x assay mixture. The pre-amplification was done in a 10 µl volume including 5.0 µl Cells Direct 2x Reaction Mix; 2.5 µl combined assay mixture, 1 µl of PBS containing the target cell [or human reference RNA (Stratagene)], 0.5 µl TE (pH 8.0), and 1 µl RT-Taq enzyme. The RT step was performed at 50°C for 15 minutes, followed by 18 cycles of amplification (95°C for 15 seconds and then 60°C for 4 minutes). Pre-amplified cDNA were diluted 5 times in TE buffer and stored at −20°C.

### Microfluidic Dynamic Arrays

TaqMan Universal Master Mix (Applied Biosystems) and 96.96 dynamic array chips, together with the NanoFlexTM 4-IFC Controller and the BioMark Real –Time PCR System (Fluidigm Corporation) were used for chip based high throughput qRT-PCR arrays, performed following the standard Fluidigm protocol [23], [24]. The chip was first primed with Krytox in the IFC Controller. Then, 5 µl sample mixtures containing 2.5µ l 2x TaqMan Universal Master Mix, 0.25 µl DA sample loading reagent (Fluidigm Corporation), and 2.25 µl preamplified cDNA were pipetted into the sample inlets. 5 µl assay mix containing 2.5 µl 20x TaqMan gene expression assay mix (Applied Biosystems) and 2.5 µl DA Assay loading reagent (Fluidigm Corporation) were pipetted into the assay inlets. The chip was then loaded and mixed in the IFC Controller. qRT-PCR reactions of the chip were performed using the BioMark Real-Time PCR System. The cycling program consisted of 10 min at 95°C followed by 40 cycles of 95°C for 15 sec and 60°C for 1 min.

### Data Analyses

C_T_ readings with Biomark software’s quality check score <0.65 or C_T_ ≥35 were treated as missing/immeasurable; otherwise, we considered the gene expressed. The following ten genes were excluded because: 1) *HGF, RPS11, RPS18,* and *RPS27A* primer sets were not used on every chip; or 2), *BMI1, EIF4E, EIF4EBP1, MED1, POU5F1 (OCT4),* and *RPLPO* produced false positives on at least one chip, showing amplification in non-template control samples (reagent mix that did not contain RNA). Samples were then screened to eliminate those with poor expression (samples had to express at least 10 genes). *UBB* was selected to represent the most robust reference gene, as reported by Popovici et al. [25]; its level of expression was associated with overall expression quality in our sample. EpCAM-captured cells from patient blood samples with *UBB* C_T_ >25 were excluded. To identify CTCs, the EpCAM-captured cells from patient blood samples were further screened: they had to express both *ACTB* and *GAPDH* reference genes, and at least one of multiple epithelial markers: *KRT7, KRT8, KRT18,* and/or *KRT19.* Cells expressing CD45, a WBC marker, were excluded.

At this stage, many more CTCs were isolated from some patients than others. To balance the analysis, at random, samples were further reduced to select exactly seven cells from each of the seven cell lines, and at most five cells per patient from the CTCs. The resulting set of cell lines and CTCs comprised the analysis set used in statistical summaries and heatmaps. To normalize the expression, we computed for each sample the mean C_T_ of the reference panel of *UBB, ACTB, and GAPDH*. To compute the normalized expression of a gene in a cell, we took the negative of the difference between the raw C_T_ expression of the gene in the sample and the mean reference level in the sample (this is the negative delta C_T_). The data was then median centered for each gene (zero represents the median expression of the gene; positive/negative values correspond to higher/lower expression respectively).

To produce heatmap images of the data, the expression values were truncated to a range of +/−3 standard deviations of the centered expression (across all genes); missing values were drawn in black. To cluster the data, first, missing values were replaced by plugging in the minimum value of −3 standard deviations, reflecting the low levels of expression that they represent. Then standard hierarchical clustering was used with the Euclidean distance metric. All analyses were performed using R software version 2.13.1 (http://cran.r-project.org/).

## Results

### Assay Validation

**Figure 2:**
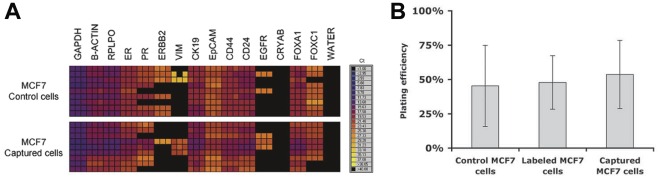
Unperturbed gene expression and cell viability of MagSweeper isolated tumor cells. A. Gene expression heat maps of C_T_ measurements of 15 genes by microfluidic qRT-PCR assays performed on single MCF7 cells before and after labeling and capture by the MagSweeper. Each gene is measured in triplicate for each single cell. Some single cell expression variation is inherent among individual cells, but the overall pattern showed no marked effect by our isolation protocol. B. Average plating efficiency (percent of single cells that formed colonies after seven days) of MCF7 cells; either control, labeled with beads, or labeled and captured by the MagSweeper, performed in triplicate. This demonstrates that cell viability was not affected by our purification protocol.

To test whether sample processing with the MagSweeper itself altered gene expression profiles, we measured the expression of a subset of 15 genes in breast cancer cell lines before and after cell processing. Overall gene expression pattern was not altered during the labeling or dynamic capture processes of our MagSweeper isolation protocol, although we noted that even within clonally-derived cell cultures before processing, some variation exists at the single cell level (Figure 2A). Moreover, the plating efficiency of cancer cell lines was similar before and after undergoing magnetic bead labeling and cell isolation, confirming no discernible effect on cell viability (Figure 2B).

We next demonstrated that high dimensional single cell analysis reliably characterizes tumor cells using 96.96 Dynamic Arrays to measure the expression of 87 cancer-associated and reference genes in individual cells isolated from primary and metastatic breast cancer cell lines. This exploratory panel of genes was selected from the published literature and our previous work in breast cancer gene expression for their role in molecular pathways relevant to breast cancer and to represent breast cancer biomarkers, prognostic markers, and phenotypes associated with cancer signaling pathways, epithelial-mesenchymal transition (EMT), cancer stem cells, and metastasis, as well as phenotypes indicative of contaminating leukocytes (Table S1).

**Figure 3:**
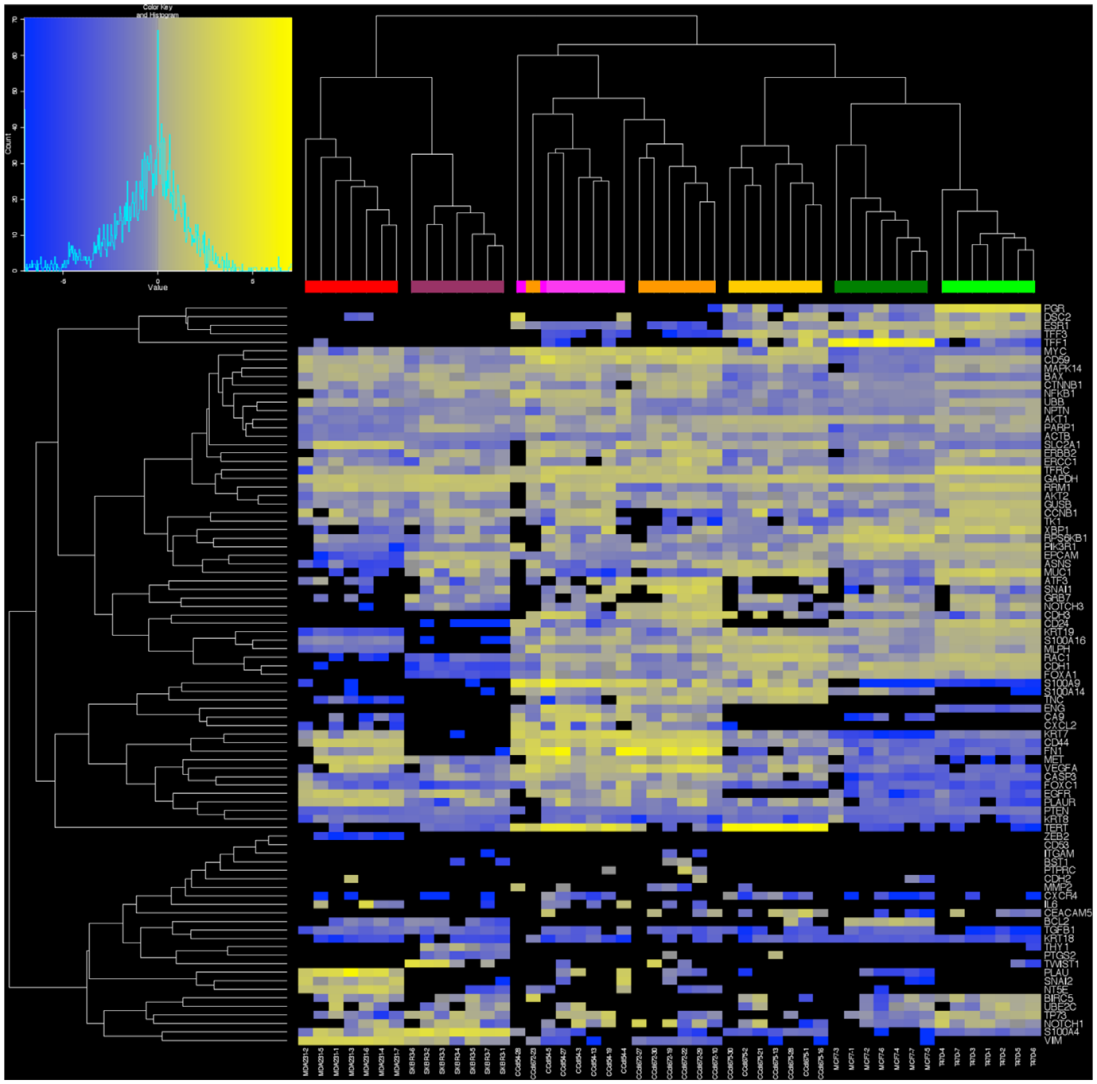
High dimensional analysis of single cells from breast cancer cell lines. A. Heatmap of single cell gene expression of 87 genes within seven individual cells isolated from three primary tumor-derived (pink: CCdl054, orange: CCdl672, gold: CCdl675), and four metastatic effusion-derived (red: MDA-231 plum: SKBR3, dark green: MCF7, and bright green: T47D) breast cancer cell lines. Yellow indicates high gene expression; gray is median expression; blue indicates low expression; and black represents undetectable expression. All cells showed expected expression patterns. The breast cancer cell lines used represent a spectrum of cell differentiation, e.g., from less differentiated and more mesenchymal/stem cell-like ER-negative (basal-like) cells (MDA-231 and SKBR3) to more differentiated ER-positive (luminal-like) cells represented by CCdl054, CCdl672, CCdl675, MCF7, and T47D.

Initially, we tested assay reproducibility for single cell high dimensional profiling on randomly selected cells from each of three primary (CCdl054, CCdl672, CCdl675) and four metastatic breast cancer cell lines (T47D, MCF7, SKBR3, and MDA-MB-231). Hierarchical clustering was performed with expression data for 87 selected genes normalized by *UBB* reference gene expression for seven single cells from each cell line. We found that 48/49 cells reproducibly clustered by cell line designation (Figure 3). Moreover, the profiles of each cell line grouping were consistent with expected biomarker patterns (e.g., ER, the human epidermal growth factor receptor 2 [HER2], and the epidermal growth factor receptor [EGFR], all important biomarkers for breast cancer prognosis and/or selection of targeted chemotherapy [26], [27]). As expected, the white blood cell marker, CD45 was not expressed by any of these epithelial cells [28]. Unsupervised clustering of the cancer cell lines separated ER-negative (MDA231, SKBR3) apart from ER-positive cell lines (CCdl054, CCdl672, CCdl675, MCF7, T47D), irrespective of their primary or metastatic origin. Our single cell expression data here comprised of an 87-gene set was robust and consistent with previous clustering patterns of these primary tumor cell lines with ER-positive metastatic cell lines derived from full scale Affymetrix array data [20].

### Control Data

We used the MagSweeper to process blood samples from 45 patients without epithelial cancer: 25 healthy volunteers and 20 lymphoma patients. None had detectable cells in the capture buffer.

### CTC Gene Expression Profiling

For cells captured from breast cancer patient blood samples, gene expression was measured in a total of 510 patient cells isolated by the MagSweeper. These represented 65 blood samples from 50 patients: 20 primary breast cancer patients without detectable metastatic disease, and 30 metastatic breast cancer patients (Table S2). In this study, we only analyzed cells that expressed three reference genes (*ACTB, GAPDH, UBB)*. To normalize gene expression, we selected the ubiquitin B (*UBB*) reference gene whose reliability as a high stability control gene for qRT-PCR has been validated in a meta-analysis of over 1700 breast cancer samples; this study also showed somewhat less stable or weaker expression of *ACTB* and *GAPDH* between different tumors [25]. Thus, we selected cells that strongly expressed *UBB* at a threshold of C_T_ <25 after pre-amplification, assuming that cells that expressed all three reference genes (*ACTB, GAPDH, UBB*) and showed highly robust expression of *UBB* are less likely to contain degraded RNA. Sixty-three percent (321/510) of the cells isolated by the MagSweeper thus qualified for further analysis. From these, we selected cells that met the following stringent criteria: 1) absent expression of the leukocyte markers *CD45*; and 2) expression of any of the following epithelial markers: *KRT7, KRT8, KRT18*, and/or *KRT19*. Among EpCAM-captured cells with non-degraded reference gene RNA, 21% also expressed detectable *CD45* transcripts; they were designated as white blood cells (WBCs) and excluded from further analysis. Overall, 60% of cells with non-degraded reference gene RNA were defined as CTCs. (summarized in Table S3). No EpCAM-labeled epithelial cells were found in the blood of healthy donors (n = 25) or of lymphoma patients (n = 20).

In the hierarchical clustering analysis of CTCs, to avoid individual patient bias, no more than 5 independent RNA samples derived from EpCAM-captured *KRT+/CD45-* cells were analyzed from the same patient. Thus, the total number of single CTC profiles inclusive of all subgroups was 105, representing 40 blood samples from 35 patients –14 with primary breast cancer, and 21 with metastatic breast cancer, and summarized by ER, PR and HER2 status (Tables S2 and S4).

Thirty-one of the 87 genes evaluated were consistently detectable in at least 15 percent of the CTCs analyzed. Aside from 3 reference genes (*ACTB, GAPDH, UBB*), the remaining 28 genes most commonly expressed in CTCs represented functional categories associated with: (1) epithelial phenotype (included in our definition of CTC) - *KRT8, KRT18, KRT19, but also CTNNB1*; (2) epithelial mesenchymal transition (EMT) - *TGFß1, FOXC1, CXCR4, NFKB1, VIM, ZEB2* **;** (3) metastasis - *S100A9, NPTN, S100A4*; (4) PI3K/AKT/mTOR pathway - *AKT1*, *AKT2, PIK3R1*, *PTEN*; (5) apoptosis – *BAX, CASP3, CD53, CD59* (6) cell proliferation - *RRM1, MAPK14*; (7) DNA repair - *PARP1*; (8) cell metabolism - *SLC2A1, TFRC*; (9) stem cell phenotype - *CD24, CD44.*

**Figure 4:**
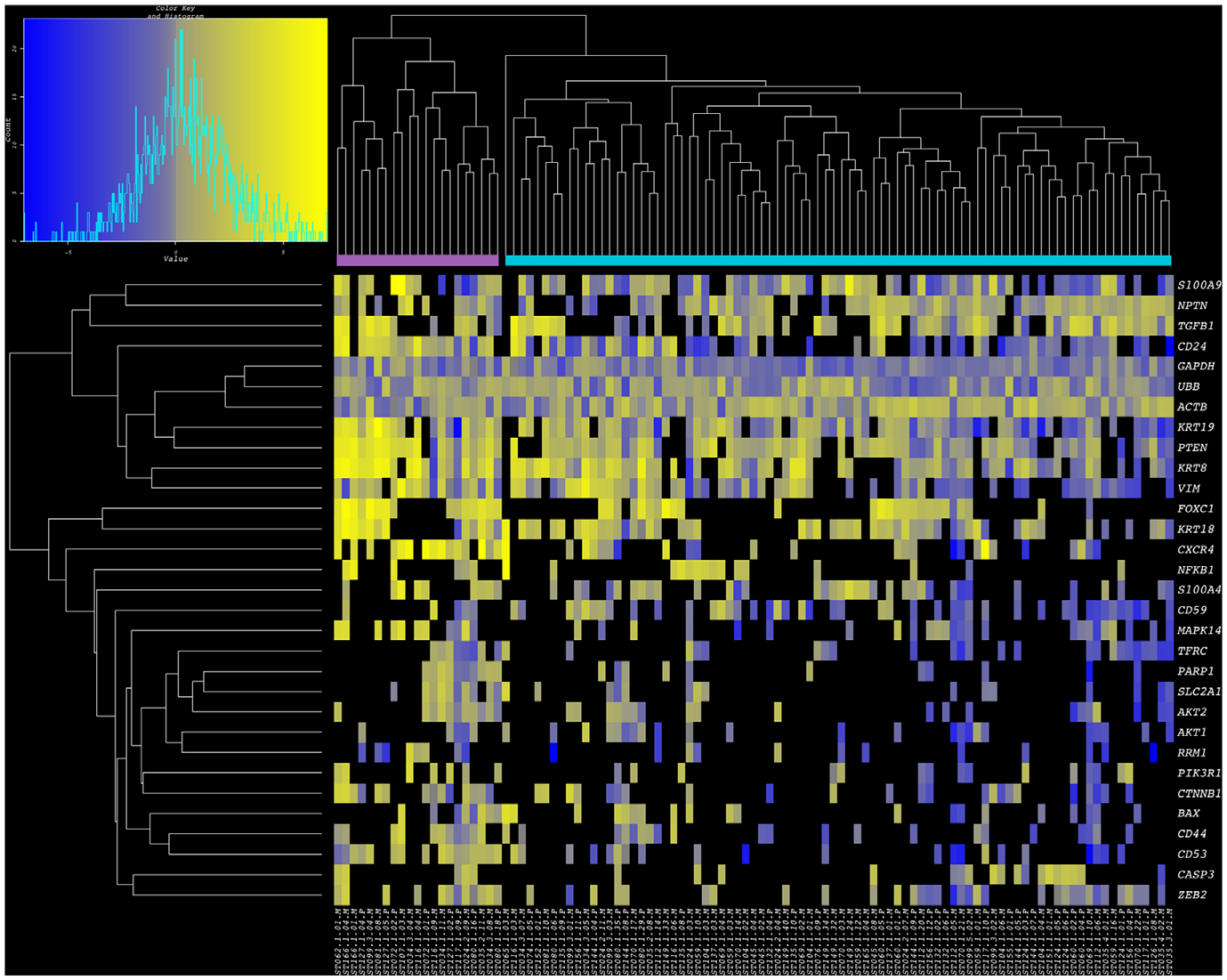
High dimensional single cell analysis and clustering of CTCs isolated from patients with breast cancer. Heatmap of single cell gene expression for 31-gene subset data derived from 105 CTCs isolated from patients with primary and metastatic breast cancer. Yellow indicates high gene expression; gray is median expression; blue indicates low expression; and black represents undetectable expression. The samples reveal two robust clusters for CTCs (lavender: Cluster I; turquoise blue: Cluster II). In addition to epithelial markers, these genes include pathways associated with EMT, metastasis, and AKT/mTOR signaling.

Unsupervised clustering analysis based on the above-mentioned subset of commonly expressed genes stratified CTCs into: (a) Cluster I - a relatively small cluster comprised of 21 cells from 13 patients, and (b) Cluster II - a larger cluster comprised of 84 cells from 30 patients (Figure 4 and Table S4). Whereas reference genes showed a similar range of variability across CTCs in both clusters, striking differences were observed for other genes. The majority of CTCs in Cluster I, as compared to CTCs in Cluster II, showed stronger expression of *S100A9, CD24, VIM, CXCR4, MAPK14, AKT2, PIK3R1, CTNNB1, CD44,* and *ZEB2*.

A final important observation was that unlike breast cancer cell lines, CTCs did not cluster by case ID. Eight (23%) cases were represented in both clusters; 5 cases were exclusive to Cluster I, and 22 cases to Cluster II (Table S4). Both clusters had similar proportions of Triple Negative, ER+, and HER2+ tumors. Differences in median patient age and disease stage (primary vs. metastatic cancer) were not significant between clusters (Table 1).

**Table 1:** Phenotype of Primary Tumors in CTC Clusters.

CTCCluster	Totalcases	Numberof CTCs	Median age at primary Dx (yrs)	Primary(%)	Metastatic(%)	ER or PR- pos(%)	HER2-pos(%)	TripleNegative (%)
I	13	21	43	4/13 (31)	9/13 (69)	6/13 (46)	2/13 (15)	5/13 (38)
II	30	84	45	12/30 (40)	18/30 (60)	12/30 (40)	5/30 (17)	13/30 (43)

### Minimal Concurrence between Profiles of CTCs and Breast Cancer Cell Lines

**Figure 5:**
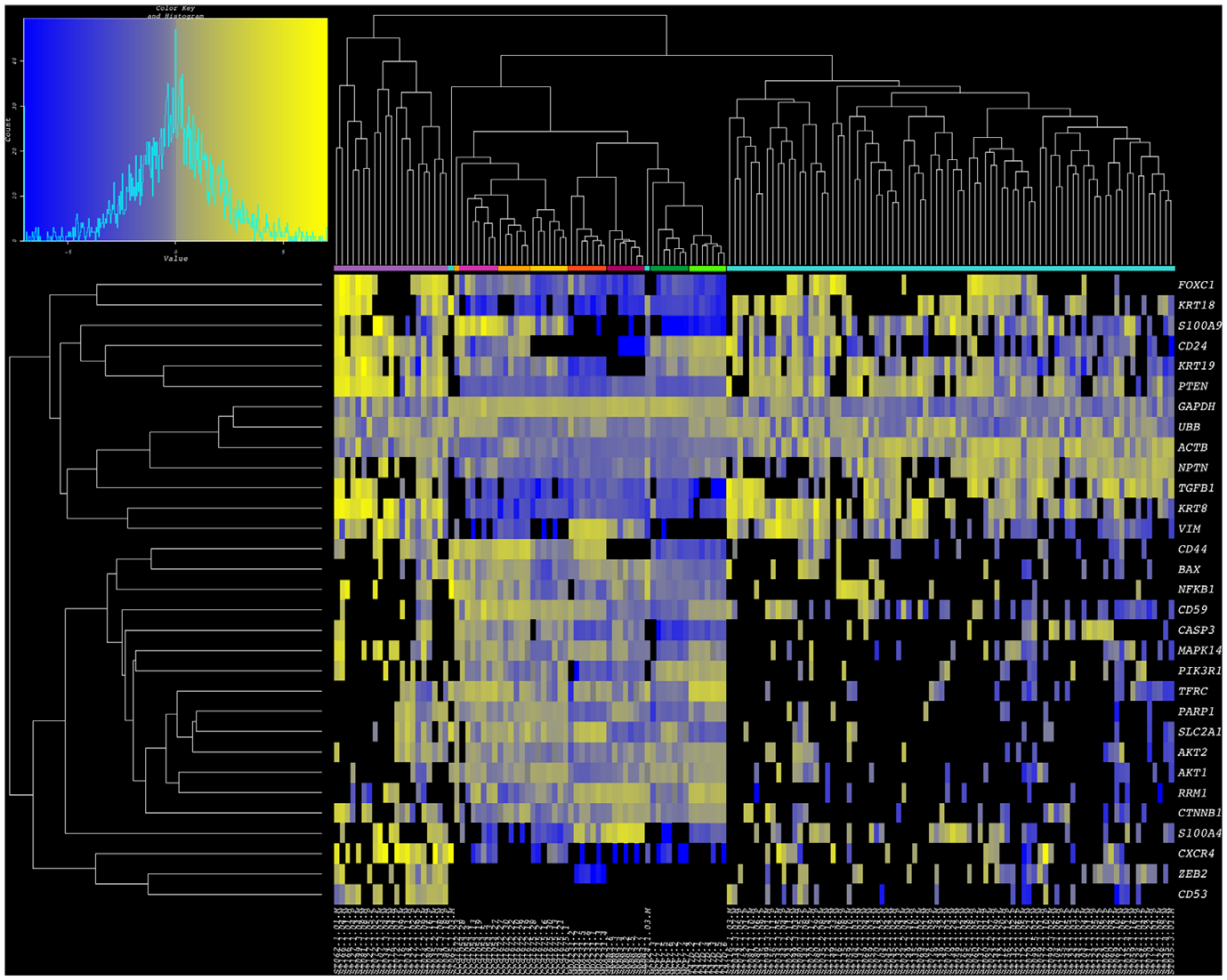
Combined breast cancer cell line and CTC clusters. Heatmap of single cell gene expression for 31-gene subset data derived from seven breast cancer cell lines and 105 CTCs isolated from patients with primary and metastatic breast cancer. Yellow indicates high gene expression; gray is median expression; blue indicates low expression; and black represents undetectable expression. The samples reveal two robust clusters for CTCs (lavender: Cluster I; turquoise blue: Cluster II) and two clusters representing primary (pink: CCdl054, orange: CCdl672, gold: CCdl675) and metastatic cell lines. Note dendrogram branches that cluster ER-negative cell lines (red: MDA-231; plum: SKBR3) and ER-positive cell lines (dark green: MCF7, and bright green: T47D).

In an effort to evaluate the similarities between widely used experimental tumor cell models and patient derived tumor cells, we combined single cell expression data from primary and metastatic breast cancer cell lines, and CTC samples towards a clustering analysis of 154 individual cells. When all 87 test genes were considered in this comparison, while cell lines and CTCs were indeed clustered apart, CTC subclassification was not robust, likely due to a large number of values resulting from undetectable transcript levels (Figure S1). However, in the analysis of 31 genes commonly expressed by CTCs, not only was intermixing of CTCs and single cells of cancer cell lines not observed (with the exception of 1/84 CTCs from Cluster II), indicating distinctive gene expression patterns of each tumor cell source, but the distinction between CTC clusters I and II was maintained relatively unperturbed. Similarly, the tumor cell lines grouped together, each with sister cells from the same culture (Figure 5). Phenotypes underlying such clustering patterns showed that CTCs maintained higher expression than all tumor cell lines for *FOXC1, KRT18, PTEN, NPTN, TGFß1, KRT8, ZEB2, and CXCR4*. On the other hand all cell lines showed elevated transcript levels for *RRM1, AKT1*, and *AKT2.* Rare similarities between experimental and clinical cell samples included elevated *VIM* expression in CTCs and ER-negative cell lines (MDA231 and SKBR3), as well as high *S100A9* expression in CTCs and ER-positive primary breast cancer cell lines (CCdl054, CCdl672, CCdl675). Overall, expression patterns of <10% (2/28) common tumor associated gene profiles of CTCs were recapitulated by a subset of tumor cell line models.

## Discussion

Over the past several years, a major factor enabling the continued characterization of surgically resected tumor tissue is the highly enriched content of malignant cells in the sample, which facilitates direct assays on primary tumor cell populations. In contrast, studying the biology of cells that successfully disseminate from the primary tumor site requires prior separation from normal components within patient blood. We have developed a cell purification technology – the MagSweeper, which gently isolates rare CTCs with high specificity. Our previous studies have shown that the MagSweeper can be used reliably to extract functional human CTCs from the blood of mice implanted with human tumor xenografts, which retain both their tumor-initiating and metastasizing capacities [17]. In conjunction with this device, here we availed recent advances in microfluidics [29], to first report on high dimensional profiling of single CTCs. We demonstrate that because our CTC isolation protocol does not impact viability and RNA integrity of isolated cells nor gene expression [16], and as evident here by consistent detection of multiple reference gene transcripts, comprehensive genomic studies on robust subpopulations of cells is greatly facilitated. Using the MagSweeper, our yield of EpCAM-labeled CTCs from patient blood samples is similar to previous reports in the literature [6], [30].

Analyzing tumor cells by their genomic and transcriptomic profiles has been an important first step towards understanding cancer biology. For example, gene expression profiling of primary tumors and its application in the molecular subtyping of breast cancer has provided a biological framework for defining the clinical heterogeneity of this disease. Although an aggressive basal breast tumor subtype was evident with select biomarkers long before the advent of genomics [31], comprehensive molecular signatures of breast cancer revealed myriad gene targets within such cancers [32]. Similarly, gene expression of disseminated tumor cells (DTCs) from bone marrow biopsies of breast cancer patients enabled clustering of different patient samples according to clinical outcome [33]. However, averaging molecular measurements across ensembles of cells [34]–[36] – as is also generally performed in expression analyses of primary or metastatic tumors [32] – obscures the granularity of individual cell biology and physiology [37]. And important for high dimensional CTC analyses (and also dependent on the particular CTC capture technique used) is addressing the issue of how to eliminate the contributions of potentially large numbers of contaminating WBCs to overall gene expression profiles when measuring genes common to both [14], [15]. Isolating CTCs from 10^7^ WBCs is a difficult problem and even techniques that enable 99.9% leukocyte depletion still show 500–1400 contaminating WBCs following enrichment [15]. In contrast, 63% of MagSweeper-captured cells showed robust, non-degraded reference gene expression: of cells with non-degraded reference RNA, 60% were defined as CTCs and 21% expressed the CD45 WBC marker. Overall, 38% of MagSweeper-captured cells (healthy and degraded) fit our robust definition of a CTC (Table S3). Although we also noted EpCAM-captured cells that expressed both cytokeratin and CD45, we eliminated these unknown cells from our CTC cluster analyses because they did not fit our stringent definition of a CTC. However, these cell types may warrant future investigation.

Single cell analysis depicts the true diversity of a heterogeneous population. We found that single CTCs displayed striking quantitative variability within a wide spectrum of genes that would have been obscured by analysis of pooled multiple cells. These analyses enabled us to identify different CTC subpopulations even within a single blood sample.

It is widely accepted that only a small minority of cells in the primary tumor are progenitors or “culprits” leading to deadly metastases. To cure cancer, such culprit cells need to be identified and characterized for targeted therapy. From the perspective of patient care, CTC biology may be more pertinent than primary tumor biology because some CTCs may follow paths to future metastatic seeding or home to specific metastatic sites. Profiling CTCs specifically refines analyses of those cells capable of entering blood vessels and surviving within the vasculature. In our study, the extracted CTCs were almost exclusively Triple Negative (lacking *ER, PR*, or *HER2* expression – Figure S1), whether or not the primary tumors of those patients displayed this phenotype (Table S2). Triple Negative tumors are aggressive and associated with higher metastatic potential, shorter time to metastasis and have limited targeted treatment options [32], [38], [39]. Others have employed CTCs in cell-by-cell qualitative studies, or low dimensional quantitative analyses for the phenotypes of this breast cancer subtype. Using indirect immunolocalization and fluorescent *in situ* hybridization (FISH), Meng et al. showed that individual tumor cells in 12 primary breast cancers, and matched CTCs either expressed HER2, urokinase plasminogen activator receptor (uPAR), both, or neither [40], exemplifying CTC heterogeneity at the single cell level. Significant discordances between ER, PR, and/or HER2 status among enriched pooled CTCs and corresponding primary tumors have been observed in patients with primary and metastatic breast cancers [41]–[45], and may lead to clinical trials testing CTC biomarkers rather than strict reliance on primary tumor biomarkers for the selection of targeted therapies. Loss of expression of *ER/PR/HER2* in CTCs noted in our particular patient samples could explain why therapies that target these biomarkers may fail to control end-stage disease; confirmation would require biopsies of late-stage metastases. Although CTC heterogeneity between patients is well recognized [46], an important finding in our study was that individual CTCs did not cluster by patient or disease stage (primary cancer vs. metastatic cancer), which again supports the concept that these cells belong to subpopulations with phenotypes fundamentally different from pooled tumor tissue, and that studying and phenotyping the primary tumor alone may lead to suboptimal treatment selection.

The demonstration of numerical/quantitative associations between CTCs and clinical outcome in previous studies [5], , albeit limited in terms of guiding molecular target based therapeutics, is indeed supportive of the hypothesis that CTCs, as a whole represent the culprit cells that lead to patient demise. Thus the simultaneous pursuit of multiple targeting strategies identified by high dimensional profiling for the elimination of all observed CTC subpopulations is warranted. Our gene expression data display CTC stratification into the major Clusters I and II, comprised of strongly and weakly expressing cells, respectively. In both clusters, robust expression of metastasis associated genes, such as *NPTN, S100A4*, and *S100A9* was striking. Notable in particular was expression of genes such as *VIM, TGFß1, ZEB2, FOXC1*, and *CXCR4*, associated with the induction and maintenance of EMT, a process by which epithelial cells transition to a more mesenchymal phenotype, both morphologically and biochemically [49]–[56], thereby increasing cell invasiveness and the link to cancer progression and poor prognosis [56], [57]. That the CTCs in Cluster II generally showed low to undetectable values for the vast majority of test transcripts suggests that characterization of this cluster could be improved further by including additional genes. Intriguingly, high levels of *PTEN* expression in 83% of the CTCs were observed despite the known inverse association between this gene and *TGFβ* expression [58], [59]. It is possible that repression of this gene by TGFβ requires receptor tyrosine kinase signaling (such as EGFR) [60], which might be compromised as indicated by undetectable *EGFR* expression in the CTCs in our study (Figure S1). Consistent with the acquisition of invasive and migratory characteristics is the absence of the cell adhesion protein, CDH1, in migrating cells [57] such as CTCs, as illustrated by our expression data. Ostensibly, systematic implementation of single cell CTC profiling will shed new light on the dynamics of migratory tumor cell biology during metastatic dissemination.

Identifying metastatic cell diversity through CTC profiling could more effectively guide drug selection in late stage cancer patients, making it reasonable to speculate that patients whose blood contains CTCs with these diverse phenotypes could greatly benefit from optimized multidrug treatment regimens. Therapy that targets only one CTC population might not ablate other subpopulations, which may continue to spread and grow. High transcript levels of genes most commonly expressed in CTCs suggest valuable targeting opportunities prior to metastatic seeding. The finding of overexpression of a metastasis-associated calcium- and zinc-binding protein encoding gene - *S100A9* [61] in CTCs suggests a valid targeting opportunity, demonstrated previously for another member of this family - S100P, in aggressive breast cancer cells [20]. As the phenotypes of CTCs continue to be revealed reliably and reproducibly in the future, it will be important to evaluate their functional response to putative druggable targets based on the biology reflected in relevant preclinical models. By including seven independent breast cancer cell line models in our single cell profiling studies, significant differences between these and CTCs could be determined. For example: (a) CTCs maintained higher expression than all tumor cell lines for *FOXC1, KRT18, PTEN, NPTN, TGFß1, KRT8, ZEB2, and CXCR4*; (b) cancer cell lines displayed measurable *CDH1* expression, but only 2/105 CTCs expressed transcripts for this epithelial cell adhesion protein that is down-regulated in EMT; and (c) all single cells within cancer cell lines of known molecular subtypes maintained *ER, PR, EGFR*, and *HER2* expression, whereas only 1, 1, 1, and 6/105 CTCs, respectively, displayed these clinically-informative phenotypes. Thus, the careful selection of appropriate experimental systems, and/or new developments will be necessary in this regard.

Our expression profiling analyses demonstrated that CTC populations are relatively quiescent. Transcript levels of growth factors and their receptors, such as *VEGFA*, *MET, ESR1, EGFR, and HER2* were relatively undetectable in CTCs compared to cancer cell lines. Consequently, expression of downstream effectors involved in cell cycle progression and proliferation such as *MYC, ATF3, TERT, RAC1, FOXA1, RRM1, CCNB1*, and *BIRC5* were significantly diminished in CTCs in contrast to breast cancer cell lines. Thus, conventional therapies targeted at proliferating cells may be inadequate for eliminating metastatic seeding by CTCs. On the other hand, we found that some CTCs maintained the expression of genes associated with the PI3K-AKT-mTOR cell survival pathway. This is significant from a clinical perspective because there are multiple new drugs under development or in early clinical trials that target this pathway [62]. By including such CTC analysis for patients entering these trials, a companion diagnostic for predicting those who may respond to these drugs could be explored. Overall, detectable variations in gene expression provide an opportunity for further fine-tuning towards more personalized approaches of targeting specific overexpressed gene products and activated pathways. Most importantly, the ease of liquid biopsies would allow optimized and timely decisions for therapeutic intervention.

## Supporting Information

Figure S1 **Cluster analysis of full multiplexed gene expression dataset in breast cancer cell lines and patient CTCs.** Heatmap of single cell expression for 87-gene profiles of 254 single cells derived from seven replicates each of seven breast cancer cell lines and 105 CTCs isolated from patients with primary and metastatic breast cancer. Yellow indicates high gene expression; blue indicates low expression; and black represents undetectable expression. The cancer cell lines (olive) cluster apart from the CTCs (brown) due to distinct differences in expression profiles. There was far greater similarity between all CTCs than with routinely used breast cancer cell lines.
(TIF)

Table S1 **Genes used to profile single CTCs.**
(DOC)

Table S2 **Patient Data.**
(DOC)

Table S3 **MagSweeper-captured single cells from breast cancer blood samples, as defined by their gene expression.**
(DOC)

Table S4 **CTC distribution in Clusters I and II.**
(DOC)
