# Supplementary figures and images for: Single Cell Profiling of Circulating Tumor Cells: Transcriptional Heterogeneity and Diversity from Breast Cancer Cell Lines
Source: PLoS One. 2012 May 7;7(5):e33788. doi: 10.1371/journal.pone.0033788 (PMC3346739; doi:10.1371/journal.pone.0033788)

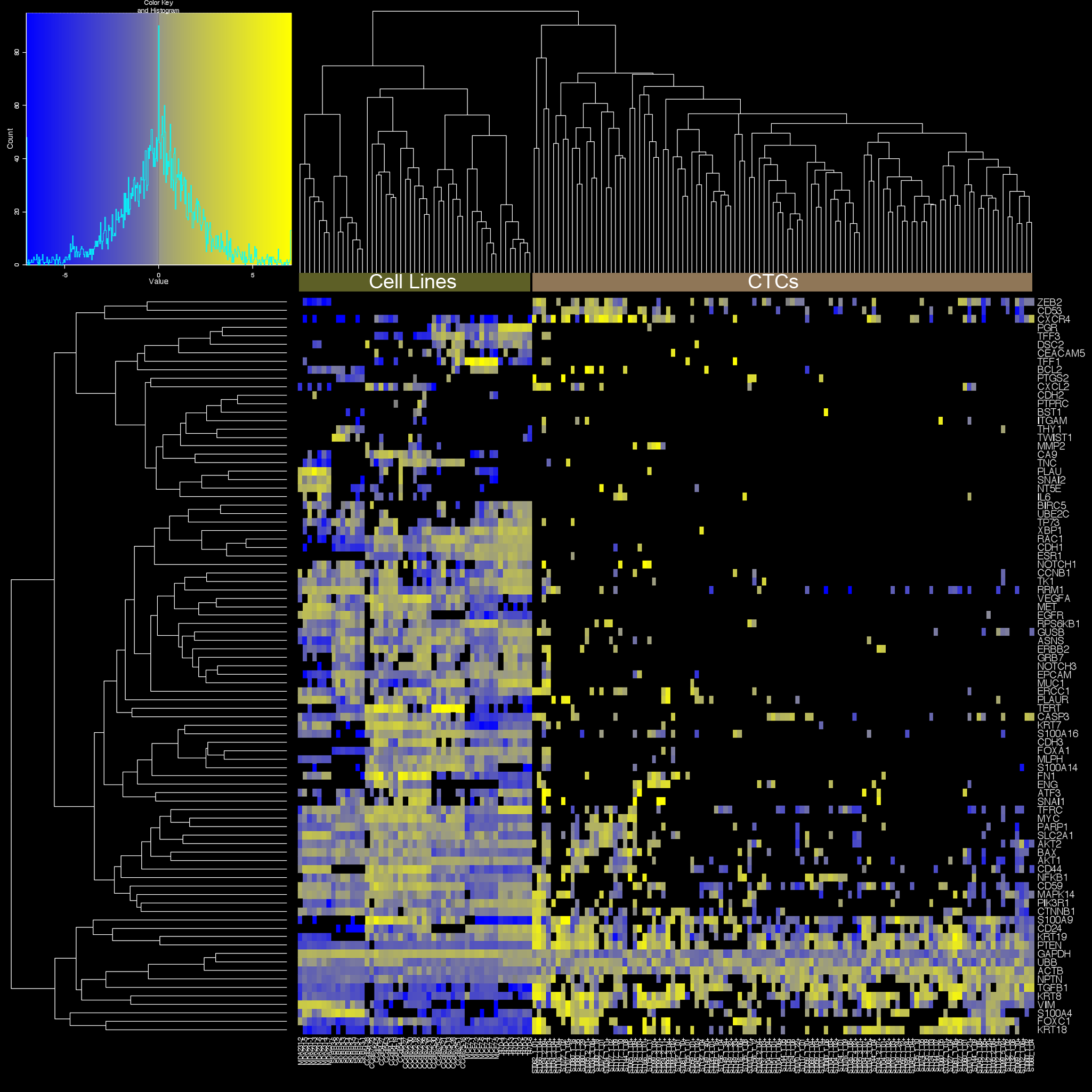

Supplement: Figure S1 — Cluster analysis of full multiplexed gene expression dataset in breast cancer cell lines and patient CTCs. Heatmap of single cell expression for 87-gene profiles of 254 single cells derived from seven replicates each of seven breast cancer cell lines and 105 CTCs isolated from patients with primary and metastatic breast cancer. Yellow indicates high gene expression; blue indicates low expression; and black represents undetectable expression. The cancer cell lines (olive) cluster apart from the CTCs (brown) due to distinct differences in expression profiles. There was far greater similarity between all CTCs than with routinely used breast cancer cell lines. (TIF) [file pone.0033788.s001.tif]
