# Supplementary material for: Single Cell Profiling of Circulating Tumor Cells: Transcriptional Heterogeneity and Diversity from Breast Cancer Cell Lines
Source: PLoS One. 2012 May 7;7(5):e33788. doi: 10.1371/journal.pone.0033788 (PMC3346739; doi:10.1371/journal.pone.0033788)
Supplement: Table S1 — Genes used to profile single CTCs. (DOC) [file pone.0033788.s002.doc]

**Table S1.** **Genes used to profile single CTCs.** Multiple 96.96 dynamic array microfluidic qRT-PCR chips used to measure the expression of 95 genes and a water control in all MagSweeper-captured single cells, non-template controls (reagent mix that did not contain RNA), and multiple dilutions of human reference RNA (Stratagene). A. Common gene set of 87 genes measured on every chip; B. Four additional primer sets used on some, but not all chips: RPS11, RPS18, RPS27A and HGF. C. Six genes that showed PCR amplification of their primers in non-template control reaction chambers on at least one chip; as false positives, these genes were excluded from all CTC analyses, even CTCs assayed on chips that did not show false-positive amplification of these genes: RPLP0; POU5F1 (OCT4); BMI1; EIF4EBP1; EIF4E; MED1.

***A. Common gene set measured in every CTC***

| ACTB | Actin, beta |
| --- | --- |
| AKT1 | V-AKT murine thymoma viral oncogene homolog 1; RAC serine/threonine protein kinase |
| AKT2 | V-AKT murine thymoma viral oncogene homolog 2; protein kinase B, beta (PKBB) |
| ASNS | Asparagine synthetase |
| ATF3 | Activating transcription factor 3 |
| BAX | BCL2-associated X protein |
| BCL2 | B-cell CLL/lymphoma 2 |
| BIRC5 | Baculoviral IAP repeat-containing protein 5; apoptosis inhibitor 4 (API4); survivin |
| BST1 | CD157; bone marrow stromal cell antigen 1 |
| CA9 | Carbonic anhydrase IX |
| CASP3 | Caspase 3, apoptosis-related cysteine protease |
| CCNB1 | Cyclin B1 |
| CD24 | CD24 antigen |
| CD44 | CD44 antigen |
| CD53 | Leukocyte surface antigen CD53 |
| CD59 | CD59 antigen; human leukocyte antigen MIC11; inhibitor of membrane attack complexes |
| CDH1 | Cadherin 1; E-cadherin; liver cell adhesion molecule (LCAM) |
| CDH2 | Cadherin 2; cadherin, neuronal (N-cadherin) |
| CDH3 | Cadherin 3; calcium-dependent adhesion protein, placental (P-cadherin) |
| CEACAM5 | Carcinoembryonic antigen-related cell adhesion molecule 5; carcinoembryonic antigen (CEA) |
| CTNNB1 | Catenin, beta-1; cadherin-associated protein, beta; beta-catenin |
| CXCL2 | Chemokine, CSC motif, ligand 2; GRO2 oncogene; macrophage inflammatory protein 2 (MIP2) |
| CXCR4 | Chemokine, CXC motif, receptor 4 |
| DSC2 | Desmocollin 2; desmocollin 3, formerly (DSC3, formerly) |
| EGFR | Epidermal growth factor receptor; HER1; ERBB1 |
| ENG | CD105; endoglin |
| EPCAM | Epithelial cellular adhesion molecule; tumor-associated calcium signal transducer (TACSTD1) |
| ERBB2 | HER2; V-ERB-B2 avian erythroblastic leukemia viral oncogene homolog 2; NEU |
| ERCC1 | Excision-repair, complementing defective, in chinese hamster, 1; UV20 |
| ESR1 | Estrogen receptor 1; estrogen receptor, alpha (ER-α) |
| FN1 | Fibronectin 1; large, external, transformation-sensitive protein (LETS) |
| FOXA1 | Forkhead box A1; hepatocyte nuclear factor 3-alpha (HNF3A) |
| FOXC1 | Forkhead box C1; forkhead, drosophila, homolog-like 7 (FKHL7) |
| GAPDH | Glyceraldehyde-3-phosphate dehydrogenase |
| GRB7 | Growth factor receptor-bound protein 7 |
| GUSB | Beta-glucuronidase |
| IL6 | Interleukin 6; interferon, beta-2 (IFNB2); B-cell stimulatory factor 2; hepatocyte stimulatory factor (HSF) |
| ITGAM | CD11B; integrin, alpha-M; complement receptor type 3, alpha subunit |
| KRT7 | Keratin 7 |
| KRT8 | Keratin 8; cytokeratin 8 |
| KRT18 | Kerain 18; cytokeratin 18 |
| KRT19 | Keratin 19 |
| MAPK14 | Mitogen-activated protein kinase 14 |
| MET | MET protooncogene; hepatocyte growth factor receptor (HGFR) |
| MLPH | Melanophilin; synaptotagmin-like protein lacking C2 domains A (SLAC2A) |
| MMP2 | Matrix metalloproteinase 2; collagenase type IV-A |
| MUC1 | Mucin 1, transmembrane |
| MYC | V-MYC avian myelocytomatosis viral oncogene homolog |
| NFKB1 | Nuclear factor kappa-B, subunit 1 |
| NOTCH1 | Notch, drosophila, homolog of, 1; translocation-associated notch homolog (TAN1) |
| NOTCH3 | Notch, drosophila, homolog of, 3 |
| NPTN | Neuroplastin; stromal cell-derived factor receptor 1 (SDFR1) |
| NT5E | CD73; ecto-5-prime nucleotidase (NT5) |
| PARP1 | Poly(ADP-ribose) polymerase 1 |
| PGR | Progesterone receptor (PR) |
| PIK3R1 | Phosphatidylinositol 3-kinase, regulatory subunit 1; p85-alpha; GRB1 |
| PLAU | Plasminogen activator, urinary; uPA; urokinase |
| PLAUR | Plasminogen activator receptor, urokinase-type; uPA receptor (UPAR) |
| PTEN | Phosphatase and tensin homolog |
| PTGS2 | Prostaglandin-endoperoxide synthase 2; cyclooxygenase 2 (COX2) |
| PTPRC | CD45; protein-tryosine phosphatase, receptor-type, C; leukocyte-common antigen |
| RAC1 | Ras-related C3 botulinum toxin substrate 1 |
| RPS6KB1 | Ribosomal protein S6 kinase, 70-kD, 1; p70-alpha; p70S6K |
| RRM1 | Ribonucleotide reductase, M1 subunit; R1 |
| S100A4 | S 100 calcium-binding protein A4; calcium placental protein; fibroblast-specific protein 1 (FSP1) |
| S100A9 | S100 calcium-binding protein A9; calgranulin B |
| S100A14 | S100 calcium-binding protein A14 |
| S100A16 | S100 calcium-binding protein A16 |
| SLC2A1 | Solute carrier family 2 (facilitated glucose transporter), member 1; glucose transporter 1 (GLUT1) |
| SNAI1 | Snail, drosophila, homolog of, 1 |
| SNAI2 | Slug; Snail, drosophila, homolog of, 2 |
| TERT | Telomerase reverse transcriptase |
| TFF1 | Trefoil factor 1; breast cancer estrogen-inducible sequence (BCEI); gastrointestinal trefoil protein pS2 |
| TFF3 | Trefoil factor 3; intestinal trefoil factor (ITF) |
| TFRC | CD71; transferrin receptor; TFR1; |
| TGFB1 | Transforming growth factor, beta-1 |
| THY1 | CD90; thy-1 T-cell antigen; |
| TK1 | Thymidine kinase, soluble |
| TNC | Tenascin C; cytotactin |
| TP73 | Tumor protein 73; p53-related protein p73 |
| TWIST1 | Twist, drosophila, homolog of 1 |
| UBB | Ubiquitin B; polyubiquitin B |
| UBE2C | Ubiquitin-conjugating enzyme E2C; UBCH10 |
| VEGFA | Vascular endothelial growth factor A |
| VIM | Vimentin |
| XBP1 | X box-binding protein 1 |
| ZEB2 | Zinc finger E box-binding homeobox 2; SMAD-interacting protein 1 (SMADIP1) |

**B. Genes measured** on some but not all chips

| *HGF* | Hepatocyte growth factor; scatter factor (SF) |
| --- | --- |
| *RPS11* | Ribosomal protein S11 |
| *RPS18* | Ribosomal protein S18 |
| *RPS27A* | Ribosomal protein S27a; ubiquitin A-80-residue ribosomal protein fusion product (UBA80); ubiquitin carboxyl extension protein 1 (UBCEP1) |

**C. Genes expressed in non-template controls (no template RNA present - false-positive result**)

| *BMI1* | Leukemia viral BMI-1 oncogene, mouse, homolog of |
| --- | --- |
| *EIF4E* | Eukaryotic translation initiation factor 4E |
| *EIF4EBP1* | Eukaryotic translation initiation factor 4E-binding protein 1; 4EBP1 |
| *MED1* | Mediator complex subunit 1; peroxisome proliferator-activated receptor-binding protein (PPARBP); PPAR-binding protein; thyroid hormone receptor interactor 2 (TRIP2) |
| *POU5F1* | POU domain, class 5, transcription factor 1; octamer-binding transcription factor 4 (OCT4); |
| *RPLP0* | Ribosomal phosphoprotein, large, P0 |
