# Supplementary material for: Single Cell Profiling of Circulating Tumor Cells: Transcriptional Heterogeneity and Diversity from Breast Cancer Cell Lines
Source: PLoS One. 2012 May 7;7(5):e33788. doi: 10.1371/journal.pone.0033788 (PMC3346739; doi:10.1371/journal.pone.0033788)
Supplement: Table S2 — Patient Data. (DOC) [file pone.0033788.s003.doc]

**Table S2. Patient Data**

|  |  |  |  | Primary Tumor Biomarkers | | |  | Metastasis Biomarkers |
| --- | --- | --- | --- | --- | --- | --- | --- | --- |
|  | Pt ID | Number of single CTCs analyzed | Primary or Metastatic CA | ER | PR | HER2 | Site of Metastases | ER/PR/HER2 |
| 1 | ST004 | 0 | M | 2+ | 3+ | 3+ | Lung, bone | neg/neg/neg |
| 2 | ST024 | 5 | M | + | + | + | Bone, eye | 1+/neg/3+ |
| 3 | ST034 | 5 | M | 3+ | neg | neg | Bone | U |
| 4 | ST035 | 5 | M | neg | neg | neg | Lung | U |
| 5 | ST040 | 0 | M | 3+ | 3+ | neg | Bone | 3+/3+/neg |
| 6 | ST041 | 0 | M | + | + | U | Chest wall | 1+/2+/neg |
| 7 | ST043 | 0 | M | U | U | U | Brain, lung | neg/neg/neg |
| 8 | ST045 | 3 | M | + | + | neg | Liver, bone | U |
| 9 | ST053 | 0 | M | + | + | + | Liver, bone | 3+/3+/neg |
| 10 | ST058 | 0 | P | neg | neg | neg | NA | NA |
| 11 | ST059 | 5 | M | neg | neg | neg | Bone, chest wall, peritoneum | U |
| 12 | ST060 | 4 | P | neg | neg | neg | NA | U |
| 13 | ST062 | 1 | M | neg | neg | U | Bone | 2+/3+/neg |
| 14 | ST065 | 1 | M | + | + | neg | Liver, lung, bone, brain | +/neg/neg |
| 15 | ST069 | 3 | M | neg | neg | pos | Liver | U |
| 16 | ST070 | 5 | M | 2+ | 3+ | neg | Bone | 3+/2+/neg |
| 17 | ST072 | 2 | P | neg | neg | neg | NA | NA |
| 18 | ST074 | 1 | P | neg | neg | neg | NA | NA |
| 19 | ST076 | 1 | P | neg | neg | neg | NA | NA |
| 20 | ST080 | 5 | P | neg | neg | neg | NA | NA |
| 21 | ST081 | 2 | M | neg | neg | pos | Liver, lung, bone, mediastinal/ retroperitoneal lymph nodes | U |
| 22 | ST099 | 5 | M | neg | 2+ | neg | Lung, bone | 3+/neg/neg (lung)  neg/U/U (bone) |
| 23 | ST104 | 5 | M | neg | neg | 3+ | Liver, lung | neg/neg/neg |
| 24 | ST107 | 1 | M | 1+ | neg | neg | Bone | neg/neg/neg |
| 25 | ST112 | 4 | M | 1+ | 3+ | neg | Brain, bone | U |
| 26 | ST113 | 0 | P | neg | neg | neg | NA | NA |
| 27 | ST116 | 4 | M | neg | neg | neg | Bone | neg/neg/neg |
| 28 | ST117 | 3 | P | neg | neg | neg | NA | NA |
| 29 | ST119 | 0 | P | + | + | neg | NA | NA |
| 30 | ST121 | 0 | P | neg | neg | neg | NA | NA |
| 31 | ST124 | 4 | P | neg | neg | neg | NA | NA |
| 32 | ST126 | 1 | M | 3+ | neg | neg | Liver, lung, bone | U |
| 33 | ST127 | 3 | P | 2+ | 2+ | neg | NA | NA |
| 34 | ST132 | 3 | P | neg | neg | neg | NA | NA |
| 35 | ST135 | 3 | P | neg | neg | neg | NA | NA |
| 36 | ST137 | 2 | M | neg | neg | neg | Lungs, bone | neg/neg/neg |
| 37 | ST140 | 0 | P | neg | neg | neg | NA | NA |
| 38 | ST144 | 5 | P | neg | neg | neg | NA | NA |
| 39 | ST149 | 5 | M | + | neg | neg | Liver | +/U/neg |
| 40 | ST150 | 0 | M | neg | neg | neg | Bone | neg/neg/neg |
| 41 | ST151 | 1 | M | U | U | U | Lung, bone | +/neg/neg |
| 42 | ST152 | 1 | P | 1+ | neg | neg | NA | NA |
| 43 | ST154 | 0 | P | neg | neg | neg | NA | NA |
| 44 | ST155 | 1 | P | 3+ | 2+ | neg | NA | NA |
| 45 | ST156 | 3 | P | neg | neg | + | NA | NA |
| 46 | ST158 | 0 | M | U | U | U | Bone | +/neg/neg |
| 47 | ST160 | 0 | M | + | + | U | Lung, bone | U |
| 48 | ST161 | 0 | M | 2+ | 3+ | neg | Liver | 2+/neg/neg |
| 49 | ST165 | 1 | M | + | + | U | Lung | U |
| 50 | ST166 | 2 | M | 3+ | 2+ | neg | Bone | +/U/neg |
|  |  | **105** |  |  |  |  |  |  |

M: metastatic breast cancer; P: primary breast cancer; U: unknown; NA: not applicable.
