# Supplementary material for: Single Cell Profiling of Circulating Tumor Cells: Transcriptional Heterogeneity and Diversity from Breast Cancer Cell Lines
Source: PLoS One. 2012 May 7;7(5):e33788. doi: 10.1371/journal.pone.0033788 (PMC3346739; doi:10.1371/journal.pone.0033788)
Supplement: Table S3 — MagSweeper-captured single cells from breast cancer blood samples, as defined by their gene expression. (DOC) [file pone.0033788.s004.doc]

**Table S3. MagSweeper-captured single cells from breast cancer blood samples, as defined by their gene expression**

|  | **Numbers of Single Cells** |
| --- | --- |
| Cells captured by MagSweeper | 510 |
| Captured “healthy” cells, defined as those without reference RNA degradation – cells expressing *ACTB (Ct <35), GAPDH (Ct<35), UBB (Ct≤25)* | 321 |
| Captured ”healthy” cells that do not express WBC marker (cells not expressing *CD45* at Ct≥35) | 252 |
| Captured “healthy” non-WBC cells that express any cytokeratin* = defined as CTCs | 194 |
| CTCs analyzed, no more than 5 randomly selected CTCs per case | 105 |

*cytokeratins = KRT7, KRT8, KRT18, KRT19

**Summary:**

1. 63% (321/510) of MagSweeper captured cells were considered “healthy.” Non-healthy cells showed RNA degradation, likely due to mitotic catastrophe/cell death or apoptosis from chemotherapy treatments and/or exposure to shear forces while circulating in the bloodstream.

2. 79% (252/321) of healthy cells captured by the MagSweeper did not express WBC markers; 21% (69/321) expressed CD45 and were considered to represent WBCs.

3. 77% (194/252) of healthy, non-WBC cells captured by the MagSweeper expressed epithelial cytokeratins.

4. 60% (194/321) of healthy cells captured by the MagSweeper were robustly defined as CTCs.

5. To avoid patient bias, particularly for patients having large numbers of CTCs and/or patients undergoing multiple blood draws during the course of their disease, CTC analyses were limited to no more than 5 per patient.
