# Supplementary material for: Single Cell Profiling of Circulating Tumor Cells: Transcriptional Heterogeneity and Diversity from Breast Cancer Cell Lines
Source: PLoS One. 2012 May 7;7(5):e33788. doi: 10.1371/journal.pone.0033788 (PMC3346739; doi:10.1371/journal.pone.0033788)
Supplement: Table S4 — CTC distribution in Clusters I and II. (DOC) [file pone.0033788.s005.doc]

**Table S4. CTC distribution in Clusters I and II**

|  | **PATIENT** | **Metastatic or**  **Primary** | **ER** | **PR** | **HER2** | **No. CTCs  analyzed per pt** | **CTCs in both  Cluster I and II** |
| --- | --- | --- | --- | --- | --- | --- | --- |
| **CLUSTER I** |  |  |  |  |  |  |  |
| 1 | ST062 | M | U | U | U | 1 |  |
| 2 | ST166 | M | pos | pos | neg | 2 |  |
| 3 | ST166R | M | pos | pos | neg |  |  |
| 4 | ST127 | P | pos | pos | neg | 3 |  |
| 5 | ST099 | M | neg | pos | neg | 5 | yes |
| 6 | ST081 | M | neg | neg | pos | 2 | yes |
| 7 | ST127R | P | pos | pos | neg |  |  |
| 8 | ST072 | P | neg | neg | neg | 2 |  |
| 9 | ST107 | M | pos | neg | neg | 1 |  |
| 10 | ST034 | M | pos | neg | neg | 5 | yes |
| 11 | ST116 | M | neg | neg | neg | 4 | yes |
| 12 | ST072R | P | neg | neg | neg |  |  |
| 13 | ST116R | M | neg | neg | neg |  |  |
| 14 | ST034R | M | pos | neg | neg |  |  |
| 15 | ST127R | P | pos | pos | neg |  |  |
| 16 | ST117 | P | neg | neg | neg | 3 | yes |
| 17 | ST024 | M | pos | pos | pos | 5 | yes |
| 18 | ST080 | P | neg | neg | neg | 5 | yes |
| 19 | ST035 | M | neg | neg | neg | 5 | yes |
| 20 | ST034R | M | pos | neg | neg |  |  |
| 21 | ST080R | P | neg | neg | neg |  |  |
| **CLUSTER II** |  |  |  |  |  |  |  |
| 1 | ST065 | M | pos | pos | neg | 1 |  |
| 2 | ST116 | M | neg | neg | neg | 4 | yes |
| 3 | ST034 | M | pos | neg | neg | 5 | yes |
| 4 | ST074 | P | neg | neg | neg | 1 |  |
| 5 | ST152 | P | pos | neg | neg | 1 |  |
| 6 | ST081 | M | neg | neg | pos | 2 | yes |
| 7 | ST080 | P | neg | neg | neg | 5 | yes |
| 8 | ST135 | P | neg | neg | neg | 3 |  |
| 9 | ST099 | M | neg | pos | neg | 5 | yes |
| 10 | ST156 | P | neg | neg | pos | 3 |  |
| 11 | ST034R | M | pos | neg | neg |  |  |
| 12 | ST144 | P | neg | neg | neg | 5 |  |
| 13 | ST024 | M | pos | pos | pos | 5 | yes |
| 14 | ST099R | M | neg | pos | neg |  |  |
| 15 | ST080R | P | neg | neg | neg |  |  |
| 16 | ST144R | P | neg | neg | neg |  |  |
| 17 | ST070 | M | pos | pos | neg | 5 |  |
| 18 | ST080R | P | neg | neg | neg |  |  |
| 19 | ST035 | M | neg | neg | neg | 5 | yes |
| 20 | ST112 | M | pos | pos | neg | 4 |  |
| 21 | ST149 | M | pos | neg | neg | 5 |  |
| 22 | ST116R | M | neg | neg | neg |  |  |
| 23 | ST135R | P | neg | neg | neg |  |  |
| 24 | ST024R | M | pos | pos | pos |  |  |
| 25 | ST059 | M | neg | neg | neg | 5 |  |
| 26 | ST104 | M | neg | neg | pos | 5 |  |
| 27 | ST137 | M | neg | neg | neg | 2 |  |
| 28 | ST069 | M | neg | neg | pos | 3 |  |
| 29 | ST059R | M | neg | neg | neg |  |  |
| 30 | ST070R | M | pos | pos | neg |  |  |
| 31 | ST104R | M | neg | neg | pos |  |  |
| 32 | ST045 | M | pos | pos | neg | 3 |  |
| 33 | ST045R | M | pos | pos | neg |  |  |
| 34 | ST132 | P | neg | neg | neg | 3 |  |
| 35 | ST024R | M | pos | pos | pos |  |  |
| 36 | ST149R | M | pos | neg | neg |  |  |
| 37 | ST135R | P | neg | neg | neg |  |  |
| 38 | ST069R | M | neg | neg | pos |  |  |
| 39 | ST104R | M | neg | neg | pos |  |  |
| 40 | ST076 | P | neg | neg | neg | 1 |  |
| 41 | ST149R | M | pos | neg | neg |  |  |
| 42 | ST149R | M | pos | neg | neg |  |  |
| 43 | ST070R | M | pos | pos | neg |  |  |
| 44 | ST149R | M | pos | neg | neg |  |  |
| 45 | ST059R | M | neg | neg | neg |  |  |
| 46 | ST165 | M | pos | pos | U | 1 |  |
| 47 | ST045R | M | pos | pos | neg |  |  |
| 48 | ST060 | P | neg | neg | neg | 4 |  |
| 49 | ST137R | M | neg | neg | neg |  |  |
| 50 | ST060R | P | neg | neg | neg |  |  |
| 51 | ST024R | M | pos | pos | pos |  |  |
| 52 | ST144R | P | neg | neg | neg |  |  |
| 53 | ST112R | M | pos | pos | neg |  |  |
| 54 | ST156R | P | neg | neg | pos |  |  |
| 55 | ST132R | P | neg | neg | neg |  |  |
| 56 | ST132R | P | neg | neg | neg |  |  |
| 57 | ST112R | M | pos | pos | neg |  |  |
| 58 | ST070R | M | pos | pos | neg |  |  |
| 59 | ST099R | M | neg | pos | neg |  |  |
| 60 | ST059R | M | neg | neg | neg |  |  |
| 61 | ST117 | P | neg | neg | neg | 3 | yes |
| 62 | ST099R | M | neg | pos | neg |  |  |
| 63 | ST104R | M | neg | neg | pos |  |  |
| 64 | ST155 | P | pos | pos | neg | 1 |  |
| 65 | ST144R | P | neg | neg | neg |  |  |
| 66 | ST124 | P | neg | neg | neg | 4 |  |
| 67 | ST144R | P | neg | neg | neg |  |  |
| 68 | ST104R | M | neg | neg | pos |  |  |
| 69 | ST151 | M | U | U | U | 1 |  |
| 70 | ST124R | P | neg | neg | neg |  |  |
| 71 | ST124R | P | neg | neg | neg |  |  |
| 72 | ST060R | P | neg | neg | neg |  |  |
| 73 | ST060R | P | neg | neg | neg |  |  |
| 74 | ST069R | M | neg | neg | pos |  |  |
| 75 | ST112R | M | pos | pos | neg |  |  |
| 76 | ST035R | M | neg | neg | neg |  |  |
| 77 | ST059R | M | neg | neg | neg |  |  |
| 78 | ST124R | P | neg | neg | neg |  |  |
| 79 | ST156R | P | neg | neg | pos |  |  |
| 80 | ST070R | M | pos | pos | neg |  |  |
| 81 | ST117R | P | neg | neg | neg |  |  |
| 82 | ST126 | M | pos | neg | neg | 1 |  |
| 83 | ST035R | M | neg | neg | neg |  |  |
| 84 | ST035R | M | neg | neg | neg |  |  |
| **Total = 105 CTCs** | R = Replicates |  |  |  |  |  |  |
